# Factors affecting women’s participation in cervical cancer screening through the theory of planned behavior: a cross- sectional study in Türkiye

**DOI:** 10.3389/fmed.2026.1910126

**Published:** 2026-08-24

**Authors:** Merve Taştan, Banu Ersoy Şen, Meltem Yılmaz

**Affiliations:** 1 Department of Family Medicine, Medical School, Amasya University, Amasya, Türkiye; 2 Directorate of Public Health Services, Amasya Provincial Health Directorate, Amasya, Türkiye

**Keywords:** early detection of cancer, health behavior, intention, theory of planned behavior, uterine cervical neoplasms

## Abstract

**Introduction:**

This study aimed to determine, within the framework of the theory of planned behavior (TPB), the psychosocial factors, such as attitude, subjective norms, perceived behavioral control, and intention, that affect participation in cervical cancer screening among women aged 30–65, as well as the mediating relationships among these factors.

**Methods:**

This descriptive cross-sectional study was conducted with 511 women aged 30–65 who applied to four Healthy Life Centers in Amasya, Türkiye between 1 April and 30 September 2025. Data were collected through face-to-face interviews using a 30-item five-point Likert-type scale based on the dimensions of TPB, along with sociodemographic questions. Descriptive statistics, *t*-tests, Pearson correlation, binary logistic regression, confirmatory factor analysis, and bootstrap mediation analysis using Hayes PROCESS were employed in the statistical analyses.

**Results:**

Among the participants, 61.3% had undergone cervical cancer screening at least once during their lifetime. The TPB subdimensions generally demonstrated good internal consistency (attitude α = 0.858; perceived behavioral control α = 0.845; intention α = 0.700; revised subjective norms α = 0.791), and four-factor model demonstrated a good fit (comparative fit index = 0.993; Tucker–Lewis index = 0.992; root mean square error of approximation = 0.086). In the logistic regression analysis, age [odds ratio (OR) = 1.10], cancer diagnosis in close surroundings (OR = 2.23), and intention score (OR = 1.55) were identified as independent determinants of screening behavior. Mediation analyses revealed that the effects of attitude, subjective norms, and perceived behavioral control on screening behavior occurred entirely through intention, with direct effects being non-significant and indirect effects being significant.

**Discussion:**

Within the context of TPB, participation in cervical cancer screening is explained not directly by attitudes and perceptions but through the intention shaped by these constructs. Intention emerges as the main determinant of screening behavior, along with age and a history of cancer in close surroundings. These findings suggest that beyond providing information, national intervention programs should be designed to focus on strengthening women’s intention to participate in screening.

## Introduction

Cervical cancer is among the most common cancers affecting women worldwide. In 2020, cervical cancer accounted for approximately 3% of all cancers worldwide, with approximately 600,000–660,000 new cases and more than 340,000 deaths (1- 4).

The theory of planned behavior (TPB) is one of the most robust psychosocial models to understand health-seeking behaviors related to preventive services such as cancer screening. Developed by Icek Ajzen, the TPB is a cognitive model that explains the underlying mechanisms within an individual’s decision to perform a specific behavior (5). According to the TPB, individuals’ attitude toward the behavior, subjective norms, and perceived behavioral controls determine their intention regarding that behavior, which serves as the main determinant of performance. Thus, according to the TPB, rather than occurring directly through these cognitive factors, behavior primarily occurs through intention (5).

In other words, as per the TPB, the strongest determinant of a woman’s participation in cervical cancer screening is her intention to perform that behavior. Studies conducted across diverse populations—including those in China, India, Ethiopia, and among Latino communities in the United States—have applied TPB constructs to predict intention and behavior regarding cervical cancer screening participation, consistently demonstrating that attitudes, subjective norms, and perceived behavioral control are significant predictors (6- 9). In this case, an individual’s attitude toward the behavior refers to the extent to which they evaluate undergoing cervical cancer screening positively or negatively. If a woman believes that “early diagnosis saves lives” and considers this outcome to be highly important for herself, she can develop a positive attitude toward screening. A study conducted with Latina women showed that the intention to participate in cervical cancer screening predicted actual participation in screening 6 months later (9). Meanwhile, subjective norms refers to the individual’s perception of whether significant others around her—such as her spouse, family, close friends, or healthcare professionals—would approve of this behavior. Social or familial pressure plays a determining role in the decision to undergo screening. Finally, perceived behavioral control refers to an individual’s perception of her self-efficacy in making a screening appointment, reaching the hospital, and coping with the difficulties of the procedure. If the woman perceives access to screening as easy and believes that she can manage this process, she is more likely to form an intention to engage in that behavior and translate that intention into action.

This study aimed to determine the factors affecting women’s participation in cervical cancer screening tests within the TPB framework. Examining the components that shape women’s screening behaviors—such as attitudes, social norms, and perceived behavioral control—will contribute to the development of interventions to increase participation in screening. In addition, by evaluating how these factors influence the provision of health services, the study aimed to contribute to the establishment of more effective health strategies.

## Materials and methods

### Study population and sample

This study employed a descriptive cross-sectional research design. Cervical cancer screening is regularly administered to the female population aged 30–65 every 5 years, in accordance with national screening protocols in Türkiye (10). The study population consisted of women aged 30–65 who applied, for any reason, to four Healthy Life Centers located in different districts of Amasya province in Türkiye between 1 April 2025 and 30 September 2025. According to TURKSTAT data from 2024, the female population aged 30–65 in Amasya, Türkiye is 79,982 (11). Assuming a 95% confidence level, a 5% margin of error, and a design effect of 1.5, the minimum sample size was calculated as 574 individuals. After data cleaning, 511 individuals were included in the study.

Women aged 30–65 who had the cognitive capacity to answer the questions and voluntarily consented to participate were included. Those who provided incomplete data or did not agree to participate were excluded. Data were collected through face-to-face interviews.

### Data collection tools

The data were collected by the researchers using a self-developed 30-question form prepared based on the subdimensions of the TPB. The form comprises five sections: sociodemographic and clinical characteristics, attitude toward the behavior, subjective norms, perceived behavioral control, and intention. To evaluate the comprehensibility and applicability of the form, a pilot test was conducted with 30 women, and the questions were revised based on their responses.

The subdimension items were evaluated and scored using a five-point Likert-type scale (1 = Strongly disagree, 5 = Strongly agree). Higher scores indicate an increased level of positive perception regarding the relevant dimension.

### Variables

The dependent variable was defined as the status of having undergone cervical cancer screening (Yes/No). The independent variables were the TPB subdimensions (attitude toward the behavior, subjective norms, perceived behavioral control, and intention), and sociodemographic and clinical variables.

### Statistical analysis

Descriptive statistics were presented as number, percentages, means, standard deviations, and median values. The conformity of the data to normal distribution was evaluated through the Shapiro–Wilk test and examining skewness-kurtosis values. The independent samples *t*-test was used for two-group comparisons. Pearson correlation analysis was applied to examine the relationships between continuous variables.

Binary logistic regression analysis was performed to determine the variables predicting the status of having undergone cervical cancer screening. The results were reported using odds ratios and 95% confidence intervals. All data analysis was performed using IBM SPSS Statistics 23.0.

### Validity and reliability analyses

To evaluate the psychometric properties of the subdimensions of the TPB, internal consistency analyses were first conducted. The Cronbach’s alpha coefficient was calculated for each subdimension, and values of 0.70 and above were considered indicative of acceptable internal consistency (12). In addition, corrected item-total correlations were examined for each item, and values above 0.30 were accepted as an indicator of adequate discriminative power. When necessary, the “alpha if item deleted” values were also evaluated, and the structural integrity of the scale was examined. The level of statistical significance was set as *p* < 0.05 in all analyses.

Confirmatory factor analysis was performed to test the scale’s construct validity. The analysis was conducted using the jamovi program version 2.7.38, using the sem module. In the measurement model, four latent variables were defined: attitude toward the behavior (six items), subjective norms (four items), perceived behavioral control (five items), and intention (three items). The items were assigned to their respective theoretical subdimensions, and covariances between factors were permitted. The dampened weighted least squares method was used for model estimation.

The evaluation of model fit considered chi-square (χ^2^), degrees of freedom (df), χ^2^/df ratio, comparative fit index (CFI), Tucker–Lewis index (TLI), normed fit index (NFI), standardized root mean square residual (SRMR), and root mean square error of approximation (RMSEA) values. A χ^2^/df value below 5 (13), CFI and TLI values above 0.90 (14, 15), an SRMR value below 0.08 (16), and an RMSEA value of 0.08 or below (17) were considered indicative of acceptable model fit.

#### Structural model analysis

Following confirmatory factor analysis, the structural model was tested in accordance with the hypothetical structure of the TPB. In this model, the effects of attitude toward the behavior, subjective norms, and perceived behavioral control on intention, as well as the effect of intention on the behavior of undergoing cervical cancer screening, were examined. Structural path coefficients, standardized beta values, and significance levels were reported.

#### Mediation analyses

The mediating role of intention in the effect of the subdimensions of the TPB on screening behavior was tested using Hayes PROCESS Procedure for SPSS Version 4.2 Macro (Model 4) with the bootstrap method (5,000 samples, 95% confidence interval). A confidence interval that did not include zero was accepted as indicating the significance of the indirect effect.

### Ethical considerations

Ethical approval for the study was obtained from the Amasya University Non-Interventional Clinical Research Ethics Committee (decision no. 2025/45) dated 20 March 2025. Permission was also obtained from the Amasya Provincial Health Directorate (letter no. 272567500) dated 27 March 2025.

## Results

The study group consisted predominantly of women who were married (89.9%), aged 30–50 (77.3%), and living in urban areas (83.8%). Approximately two-thirds of the participants had undergone cervical cancer screening at least once in their lifetime (61.3%). More than half had undergone screening for another cancer (50.5%), and 46.8% had experience with cancer in their close surroundings (Table 1).

**TABLE 1:** Sociodemographic and clinical characteristics of participants.

Variables	*n*	%
Marital status		
Married	455	89.0
Single	56	11.0
Age		
30–40	221	43.2
41–50	174	34.1
51–60	78	15.3
61–65	38	7.4
Settlement		
Rural	83	16.2
Urban	428	83.8
Education level		
Primary school	130	25.4
Middle school	13	2.5
High school	109	21.3
Associate’s degree	58	11.4
Bachelor’s degree	168	32.9
Master’s degree and above	33	6.5
Employment Status		
Employed	266	52.1
Unemployed	245	47.9
Occupation		
Homemaker	218	42.7
Health professional	161	31.5
Education sector	46	9.0
Public sector employee	42	8.2
Private sector employee	25	4.9
Retired	10	2.0
Others	7	1.4
Presence of chronic disease		
Yes	125	24.5
No	386	75.5
Cervical cancer screening status		
Yes	313	61.3
No	198	38.7
Other cancer screening status		
Yes	258	50.5
No	253	49.5
Family history of cancer		
Yes	206	40.3
No	305	59.7
Cancer diagnosis in close social circle		
Yes	239	46.8
No	272	53.2

The internal consistency analyses of the subdimension items developed for TPB indicated that the subdimensions generally demonstrated acceptable to good levels of reliability. The Cronbach’s alpha of the intention subdimension was 0.700, indicating an acceptable level. The perceived behavioral control subdimension showed high internal consistency (α = 0.845). Attitude toward the behavior had the highest reliability (α = 0.858). In the initial analysis of subjective norms, the Cronbach’s alpha coefficient was 0.547. One item with a very low corrected item-total correlation (*r* = 0.012) was removed from the scale. Subsequently, the Cronbach’s alpha coefficient increased to 0.791, indicating that internal consistency had reached a good level. Corrected item-total correlations were generally within acceptable limits; apart from the removed item in the subjective norms subdimension, deleting any other item from the scale did not result in a considerable increase in the alpha coefficient. Therefore, along with the revised subjective norm subdimension, all remaining items were included in the analyses (Table 2).

**TABLE 2:** Item analysis and internal consistency of the theory of planned behavior subscales.

Subscale	Item	Corrected item–total correlation	Cronbach’s alpha if item deleted	Cronbach’s alpha
Intention (INT)	INT1	0.588	0.589	0.700
	INT2	0.621	0.504	
	INT3	0.471	0.792	
Perceived behavioral control (PBC)	PBC1	0.657	0.813	0.845
	PBC2	0.758	0.783	
	PBC3	0.666	0.814	
	PBC4	0.698	0.802	
	PBC5	0.508	0.849	
Subjective norm (SN) – revised	SN1	0.471	0.408	0.791
	SN2	0.534	0.379	
	SN3	0.466	0.455	
	SN4	0.454	0.412	
Attitude toward the behavior (ATB)	ATB1	0.568	–	0.858
	ATB2	0.580	–	
	ATB3	0.824	–	
	ATB4	0.681	–	
	ATB5	0.584	–	
	ATB6	0.612	–	

One item in the subjective norm subscale was removed due to a low corrected item-total correlation (*r* = 0.012), which increased the subscale’s Cronbach’s alpha coefficient from 0.547 to 0.791.

**FIGURE 1:**
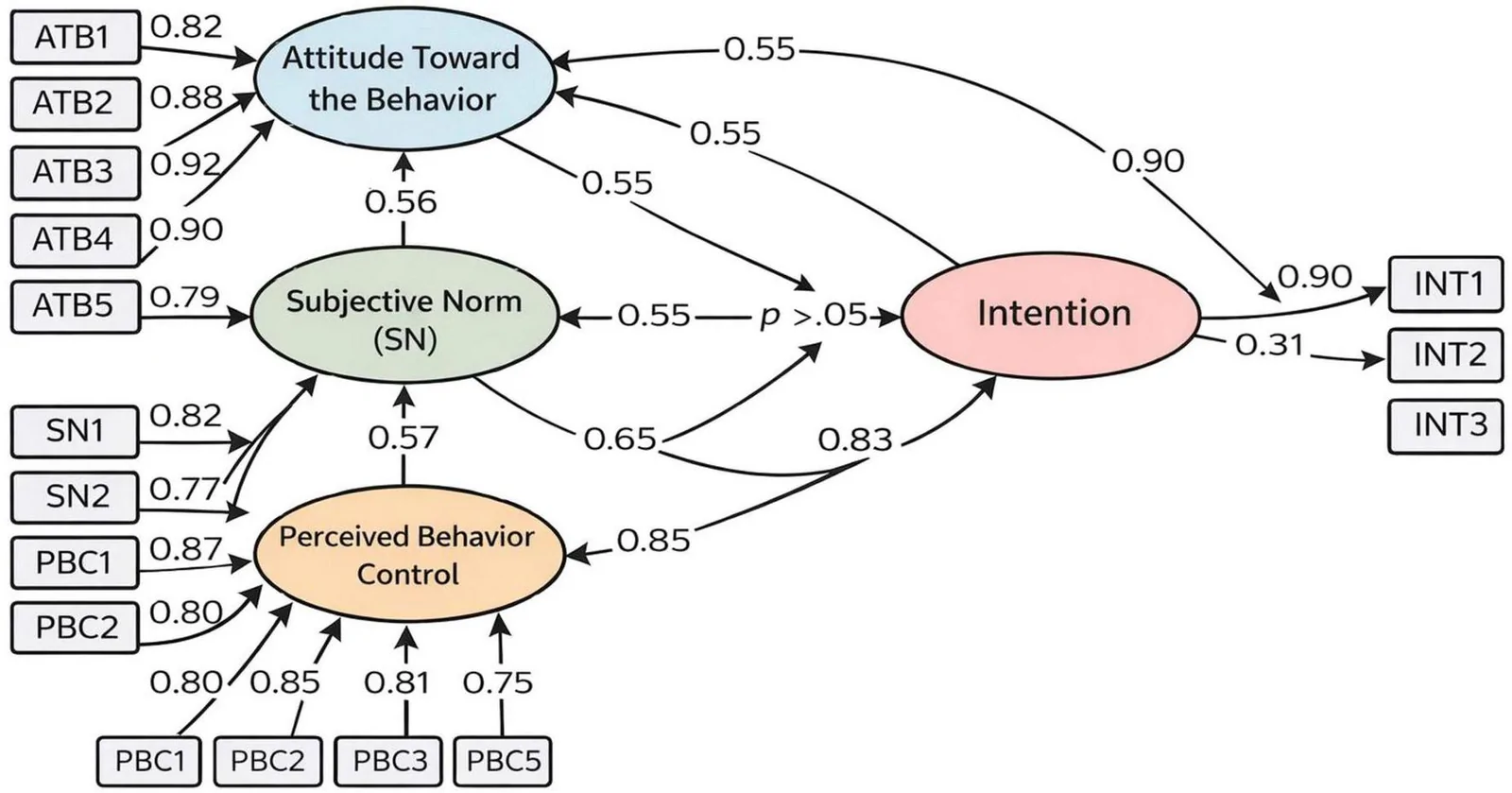
Confirmatory factor analysis diagram of the measurement model based on the theory of planned behavior.

Confirmatory factor analysis indicated that the fit of the four-factor measurement model to the data was good (χ^2^ = 621, df = 129, *p* < 0.001). All values of the fit indices (CFI = 0.993, TLI = 0.992, IFI = 0.993, SRMR = 0.058) indicated good model fit. The RMSEA value was found to be 0.086, which falls within acceptable fit limits. The factor loadings of all items on their respective latent variables ranged from 0.749 to 0.919, and each item was statistically significant (*p* < 0.001). These findings support the construct validity of the measurement model (Table 3). In the model, the latent variables of attitude, subjective norms, perceived behavioral control, and intention are represented by the relevant observed variables. The coefficients in Figure 1 indicate the standardized factor loadings and the relationships among the latent variables.

**TABLE 3:** Confirmatory factor analysis fit indices of the research scale.

Model	χ^2^	df	χ^2^/df	RMSEA	SRMR	CFI	TLI
Four-factor measurement model	621	129	4.81	0.086	0.058	0.993	0.992

RMSEA, root mean square error of approximation; SRMR, standardized root mean square residual; CFI, comparative fit index; TLI, Tucker-Lewis index.

The intention scores for undergoing screening among married participants (4.15 ± 0.89) were statistically significantly higher than those of single participants (3.85 ± 0.94) (*p* = 0.016). Similarly, the intention scores of employed individuals were higher than those of unemployed individuals (*p* = 0.044). As age increased, scores for perceived behavioral control (*p* = 0.008) and attitude (*p* = 0.022) decreased. In contrast, as education level increased, individuals’ belief in their ability to control their behavior (*p* = 0.031) and positive attitudes (*p* = 0.003) increased. Participants living in urban areas scored significantly higher than those living in rural areas across all subdimensions of the TPB. Moreover, the intention, perceived behavioral control, and attitude scores of healthcare workers were significantly higher than those employed in non-health sectors.. The intention to undergo screening (*p* = 0.007) and attitude (*p* = 0.003) were higher among individuals who had someone diagnosed with cancer in their close surroundings. However, a family history of cancer did not create a significant difference in any subdimension (*p* > 0.05). The intention and perceived behavioral control scores of individuals who had previously undergone screening were considerably higher than those of individuals who had not (*p* < 0.001) (Table 4).

**TABLE 4:** Comparison of the theory of planned behavior subscale scores by sociodemographic and clinical variables.

Variable	Group	Intention (mean ± SD)	*P*	Control (mean ± SD)	*P*	Subjective norm (mean ± SD)	*P*	Attitude (mean ± SD)	*P*
Marital status^1^	Married	4.15 ± 0.89	**0.016**	4.38 ± 0.66	0.066	3.93 ± 0.58	0.215	4.46 ± 0.56	0.149
	Single	3.85 ± 0.94	–	4.20 ± 0.61	–	3.82 ± 0.64	–	4.34 ± 0.63	–
Age^2^	–	*r* = −0.077	0.083	*r* = −0.119	0.008	*r* = −0.070	0.118	*r* = −0.102	0.022
Settlement^1^	Rural	3.88 ± 1.02	**0.008**	4.20 ± 0.63	**0.017**	3.76 ± 0.55	**0.011**	4.24 ± 0.54	**<0.001**
	Urban	4.16 ± 0.86	–	4.39 ± 0.66	–	3.94 ± 0.59	–	4.48 ± 0.56	–
Chronic diseases^1^	Yes	4.09 ± 0.96	0.676	4.33 ± 0.71	0.579	3.90 ± 0.61	0.685	4.42 ± 0.57	0.546
	No	4.13 ± 0.88	–	4.37 ± 0.64	–	3.92 ± 0.58	–	4.45 ± 0.57	–
Employement status^1^	Employed	4.19 ± 0.86	**0.044**	4.41 ± 0.64	0.080	3.95 ± 0.59	0.135	4.49 ± 0.56	0.052
	Unemployed	4.03 ± 0.93	–	4.30 ± 0.67	–	3.87 ± 0.59	–	4.39 ± 0.57	–
Occupation^1^	Health professional	4.24 ± 0.83	**0.033**	4.48 ± 0.65	**0.007**	3.97 ± 0.60	0.125	4.54 ± 0.55	**0.011**
	Non-health professional	4.06 ± 0.93	–	4.31 ± 0.66	–	3.89 ± 0.59	–	4.40 ± 0.57	–
Education level^2^	–	*r* = 0.087	0.051	*r* = 0.111	**0.031**	*r* = 0.082	0.068	*r* = 0.131	**0.003**
Cancer diagnosis in close social circle^1^	Yes	4.23 ± 0.84	**0.007**	4.39 ± 0.68	0.360	3.95 ± 0.58	0.263	4.52 ± 0.52	**0.003**
	No	4.02 ± 0.94	–	4.33 ± 0.63	–	3.89 ± 0.60	–	4.37 ± 0.60	–
Family history of cancer^1^	Yes	4.15 ± 0.91	0.460	4.38 ± 0.69	0.452	3.96 ± 0.61	0.150	4.46 ± 0.59	0.575
	No	4.09 ± 0.89	–	4.33 ± 0.63	–	3.88 ± 0.57	–	4.43 ± 0.55	–
Other cancer screening status^1^	Yes	4.28 ± 0.89	**<0.001**	4.47 ± 0.62	**<0.001**	3.96 ± 0.58	0.116	4.50 ± 0.58	**0.031**
	No	3.95 ± 0.87	–	4.25 ± 0.67	**–**	3.87 ± 0.60	–	4.39 ± 0.55	–
Cervical cancer screening status^1^	Yes	4.28 ± 0.84	**<0.001**	4.46 ± 0.59	**<0.001**	3.94 ± 0.55	0.215	4.48 ± 0.56	0.086
	No	3.85 ± 0.93	–	4.20 ± 0.72	–	3.87 ± 0.65	–	4.39 ± 0.57	–

^1^The independent samples *t*-test was used for comparisons between groups.

^2^Pearson correlation analysis was conducted. Statistically significant coefficients and estimates (*p* < 0.05) are shown in bold.

Binary logistic regression analysis was performed to determine the independent effects of variables showing a significant relationship with screening behavior, and age, having a cancer diagnosis in one’s close surroundings, and intention were identified as the strongest independent determinants of screening behavior. Each one-unit increase in age increased the individual’s likelihood of undergoing screening by 1.10 times (*p* < 0.001). The probability of undergoing screening among individuals who had a cancer diagnosis in their close surroundings was 2.23 times higher than among those who did not (*p* < 0.001). Finally, each one-unit increase in the individual’s intention score increased the probability of performing screening behavior by 1.55 times (*p* = 0.015). Other variables such as marital status, presence of chronic disease, education level, and perceived behavioral control were not found to significantly determine the behavior independently within this multivariable model (*p* > 0.05) (Table 5).

**TABLE 5:** Binary logistic regression analysis of the predictors of cervical cancer screening status.

Variables	B	S.E.	Wald	*P*	OR	%95 GA
Year	0.097	0.014	46.740	**<0.001**	**1.102**	1.072–1.133
Marital status	0.437	0.335	1.704	0.192	1.549	0.803–2.986
Chronic diseases	−0.084	0.271	0.096	0.757	0.920	0.541–1.563
Cancer diagnosis in close social circle	0.801	0.221	13.115	**<0.001**	**2.229**	1.444–3.439
Education level	0.015	0.072	0.044	0.834	1.015	0.882–1.168
Intention	0.440	0.181	5.950	**0.015**	**1.553**	1.090–2.213
Perceived behavioral control	0.431	0.246	3.062	0.080	1.538	0.950–2.492

Statistically significant coefficients and estimates (*p* < 0.05) are shown in bold.

**FIGURE 2:**
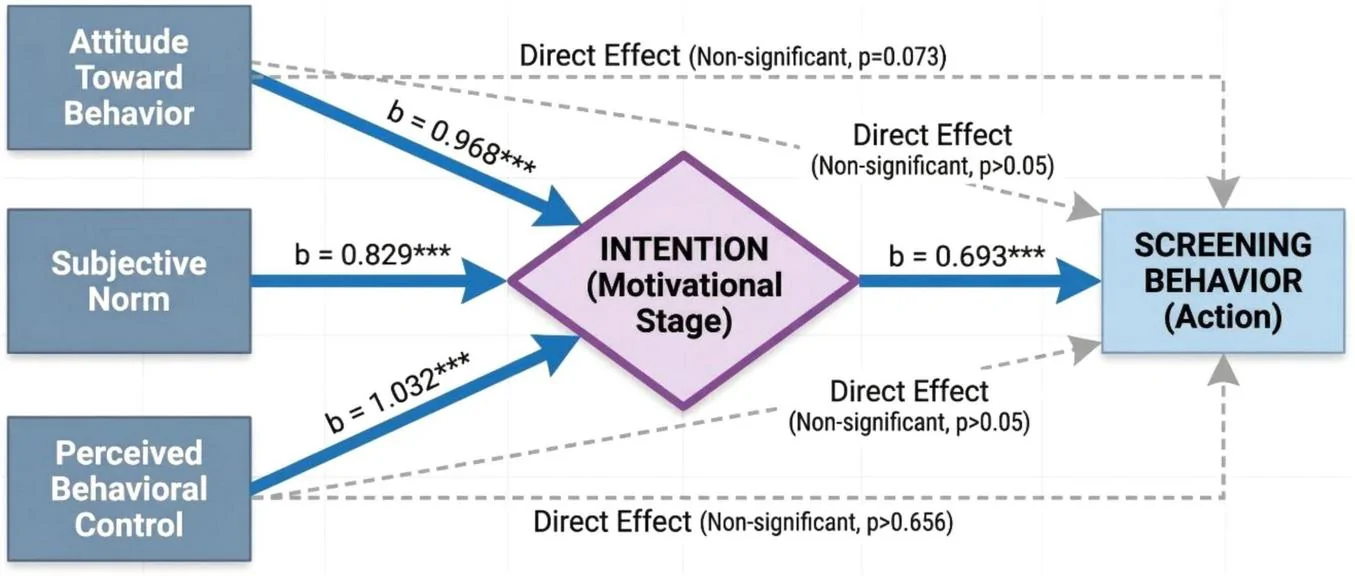
Path model of the theory of planned behavior and the full mediating role of intetion. ***P < 0.001. Direct effects are statistically non-significant.

Bootstrap mediation analyses were used to examine the mediating role of intention in the relationship between attitude toward the behavior, subjective norms, and perceived behavioral control and screening behavior. Attitude toward the behavior had a significant effect on intention (b = 0.968, *p* < 0.001), and intention also had a significant effect on screening behavior (b = 0.693, *p* < 0.001). Meanwhile, the direct effect of attitude toward the behavior was non-significant (*p* = 0.073). The 95% bootstrap confidence interval for the indirect effect ranged from 0.392 to 1.053, and it did not include zero. The effect of subjective norms on intention was significant (b = 0.829, *p* < 0.001), but its direct effect on screening behavior was not significant (*p* > 0.05). The 95% confidence interval of its indirect effect through intention ranged from 0.344 to 0.851 and did not include zero. The effect of perceived behavioral control on intention was significant (b = 1.032, *p* < 0.001), and the effect of intention on screening behavior was significant (b = 0.517, *p* = 0.0017). However, the direct effect was not significant (*p* = 0.656), and the 95% confidence interval for the indirect effect ranged from 0.186 to 0.938 and did not include zero. In all models, the direct effects were non-significant, whereas the indirect effects were significant. These findings indicate full mediation (Figure 2).

## Discussion

This study examined women’s cervical cancer screening behavior within the framework of the TPB and yielded findings that strongly support the theory’s structural model. The most noteworthy result is that attitude toward the behavior, subjective norms, and perceived behavioral control did not directly affect screening behavior, and their effect occurred entirely indirectly through “intention.”

The participants’ scores in the TPB subdimensions indicated that general attitudes toward screening behavior and perceived behavioral control were quite high. Similarly, in Ethiopia, women applying to maternal and child health services had relatively high scores in attitude and perceived behavioral control, as measured by TPB; all subdimensions of TPB were positively associated with intention, with perceived behavioral control identified as the strongest determinant (18).

The fact that the scores for subjective norms remained relatively lower than those of the other dimensions suggests that, in the decision to undergo screening, the individual’s own cognitive evaluation takes precedence over the expectations of the social environment (e.g., family, friends, spouse). Recent studies conducted in Türkiye support the view that the greatest barrier in women’s attitudes toward cervical cancer screening is lack of information and motivation; as positive attitude develops, the need for external approval (social norm) decreases (19). Studies conducted in Türkiye demonstrate that participation in cancer screening is generally low, with lack of information and emotional barriers representing important determinants (20). In a study conducted in Hossana, Ethiopia insufficient knowledge was found to be closely associated with negative attitudes and low screening rates (21). Other studies from Türkiye, especially those involving migrant or Turkish women, indicate that as knowledge and motivation increase, positive attitude becomes stronger; thus the need for family approval may become relatively less important. Screening behavior relies more on the individual’s own risk perception and evaluation of benefits (20, 22–24).

In this study, the scores for intention, perceived control, and attitude were higher among healthcare workers, demonstrating the effect of “occupational group” on screening behavior, which has not been sufficiently elaborated in previous studies. It has been shown that health behaviors vary according to profession and working conditions among health professionals such as nurses. In a study conducted in Poland, the overall health behavior levels of nurses were found to be moderate; however, occupational factors such as workplace type and duration of experience led to significant differences in areas such as nutrition and mental attitude (25). This finding also supports the view that healthcare workers exhibit a distinct health behavior profile due to their profession. As the occupational dimension has not been addressed in such detail in most cancer screening studies, presenting healthcare workers as a separate psychosocial profile, especially by quantitatively distinguishing them through TPB constructs, offers a detailed and original contribution to the literature (18, 26, 27).

In this research, age and having a cancer diagnosis in close surroundings predicted screening behavior independently of intention. When the factors determining screening behavior were examined through the logistic regression model, age, history of cancer in close surroundings, and intention were identified as the strongest independent variables determining the behavior. The literature also documents the increase in participation in cancer screening with advancing age (28- 31). Individuals who had someone diagnosed with cancer in their close surroundings were 2.23 times more likely to undergo screening, emphasizing the critical role of the perceived sense of risk formed in the individual in initiating action. Personal or familial cancer experience increases risk perception and defensive motivation and promotes screening by prompting action. Other studies consistently report that having a history of cancer in oneself, one’s family, or one’s close surroundings is a trigger that strengthens screening behavior (30- 33). On the other hand, perceived behavioral control and attitude scores decreased with increasing age.

This suggests that, even though women aged 30–65 maintain their belief in the benefits of the screening program as they age, they perceive the act of undergoing screening as more difficult and complex. As women age, postmenopausal physical discomfort and the belief that screening is no longer necessary negatively affect their participation in cervical cancer screening (34). However, it has been demonstrated that the risk of cancer among those who undergo testing is reduced by one-quarter compared to those who do not, and that even receiving an abnormal result offers a protective effect against cancer relative to those who do not get tested (35).

Moreover, this study found that those living in urban areas, are employed, and with higher levels of education had higher scores in the bivariate analyses, confirming the protective effect of health literacy and ease of access to health services—especially Cancer Early Diagnosis, Screening and Education Centers—on preventive health behaviors in Türkiye. In Türkiye and other countries, living in urban areas, having a higher level of education, and participating in working life are identified as the main factors increasing health literacy (36, 37) and the use of preventive services (38, 39). The lower perceived behavioral control among women living in rural areas supports the barriers reported in the literature, such as transportation difficulties, privacy concerns, and inability to find time (40, 41).

In most TPB-based screening studies, perceived behavioral control and intention directly and indirectly affect behavior through intention; that is, the typical pattern is partial mediation (18, 27, 42). The most original and noteworthy finding of our study emerged from the mediation analyses. Although some studies in the international literature report that perceived behavioral control directly affects screening behavior, our study identified a full mediation effect. In other words, individuals’ positive attitudes toward screening behavior, feelings of social pressure, and beliefs in their ability to perform the behavior do not translate directly into the action. Rather, the effects of attitude, subjective norms, and perceived behavioral control on screening behavior operated entirely through the mediating variable of intention, with no direct effect. This result distinguishes this study from the literature. Studies examining organic food purchasing, exercise, and oral health behaviors have shown that intention and perceived behavioral control predict behavior not through full mediation but through partial mediation (43- 45). The identification of full mediation in screening behavior represents a rare and methodologically noteworthy result, particularly for the TPB literature.

In this study, one item in the subjective norm subdimension was excluded from the analysis to ensure statistical reliability because it exhibited a low item-total correlation (*r* = 0.012). Subjective norms measure an individual’s perception of whether a behavior is approved by the social environment that the individual takes as a reference. As the behavior in question, “cervical cancer screening,” is a procedure that directly involves gynecological and bodily privacy, women in Turkish society tend to evaluate this decision not according to the expectations of their broader social environment but as a more internal and private process. Women perceive gynecological examination not as a “social issue” to be submitted to the approval of their extended family or friend circle but as a private health right. This may have disrupted the compatibility of general statements measuring external pressure within the scale. This finding is noteworthy in that it shows that traditional social approval dynamics may function differently than expected in health behaviors that require privacy.

Cervical cancer screening is not a behavior that occurs spontaneously; it is a planned action that requires making an appointment, going to a healthcare institution, and being psychologically prepared for a gynecological examination. Therefore, it is not sufficient for a woman to theoretically consider screening to be beneficial or to know that cancer screening centers are free of charge; these cognitive elements must combine and transform into a strong stage of motivational readiness, namely, intention. The behavior only occurs when this bridge of intention has been crossed.

## Conclusion

This study demonstrates that screening behavior is associated not with direct cognitive evaluations but with the intention formed by these evaluations, confirming that the TPB provides a highly valid explanatory framework for this effect in a sample of Turkish women. In national cancer screening programs, direct calls to action such as “have your cervical cancer screening” remain insufficient in primary care family health centers and cancer screening centers. Cervical cancer screening is not an instantaneous act, but a planned and difficult action; for this reason, it requires intention. As intention is the absolute precondition of behavior, healthcare workers should focus on interventions that will strengthen women’s intention. Emphasizing that the procedure is painless and brief, and explaining that services are free and accessible, will strengthen the pool of intention and directly increase final screening behavior.

## Data Availability

The raw data supporting the conclusions of this article will be made available by the authors, without undue reservation.

## References

[1] Singh D Vignat J Lorenzoni V Eslahi M Ginsburg O Lauby-Secretan B et al. Global estimates of incidence and mortality of cervical cancer in 2020: a baseline analysis of the WHO global cervical cancer elimination initiative. *Lancet Global Health.* (2023) 11:e197–206. 10.1016/S2214-109X(22)00501-0 36528031 PMC9848409

[2] Sung H Ferlay J Siegel R Laversanne M Soerjomataram I Jemal A et al. Global cancer statistics 2020: GLOBOCAN estimates of incidence and mortality worldwide for 36 cancers in 185 countries. *CA Cancer J Clinicians.* (2021) 71:209–49. 10.3322/caac.21660 33538338

[3] Bray F Ferlay J Soerjomataram I Siegel R Torre L Jemal A. Global cancer statistics 2018: GLOBOCAN estimates of incidence and mortality worldwide for 36 cancers in 185 countries. *CA Cancer J Clin.* (2018) 68:394–424. 10.3322/caac.21492 30207593

[4] Li Z Liu P Yin A Zhang B Xu J Chen Z et al. Global landscape of cervical cancer incidence and mortality in 2022 and predictions to 2030: the urgent need to address inequalities in cervical cancer. *Int J Cancer.* (2025) 157:288–97. 10.1002/ijc.35369 40026066

[5] Ajzen I. The theory of planned behavior. *Organ Behav Hum Decis Process.* (1991) 50:179–211. 10.1016/0749-5978(91)90020-T

[6] Xin T Jiang Y Li C Ding X Zhu Z Chen X. Using planned behavior theory to understand cervical cancer screening intentions in Chinese women. *Front Public Health.* (2023) 11:1063694. 10.3389/fpubh.2023.1063694 36923041 PMC10008845

[7] Eshetu H Shitu K Handebo S. Predictors of intention to receive cervical cancer screening among commercial sex workers in Gondar city, northwest Ethiopia: application of the theory of planned behavior. *BMC Women’s Health.* (2022) 22:462. 10.1186/s12905-022-02055-8 36404309 PMC9830899

[8] Dsouza J Broucke S Pattanshetty S Dhoore W. A comparison of behavioural models explaining cervical cancer screening uptake. *BMC Womens Health.* (2022) 22:235. 10.1186/s12905-022-01801-2 35710374 PMC9204900

[9] Roncancio A Ward K Sanchez I Cano M Byrd T Vernon S et al. Using the theory of planned behavior to understand cervical cancer screening among latinas. *Health Educ Behav.* (2015) 42:621–6. 10.1177/1090198115571364 25712240 PMC4932857

[10] Republic of Turkey Ministry of Health. *Cancer Screenings.* (2025). Available online at: https://hsgm.saglik.gov.tr/tr/kanser-taramalari.html (accessed July 24, 2026).

[11] Turkish Statistical Institute. *Population by Province, Single Age and Sex 2025.* Available online at: https://veriportali.tuik.gov.tr/tr/search?q=ya%C5%9F+ve+cinsiyet&level=Amasya (accessed May 1, 2025).

[12] Nunnally J Bernstein I. *Psychometric Theory.* 3rd ed. New York: McGraw-Hill (1994).

[13] Wheaton B Muthen B Alwin D Summers G. Assessing reliability and stability in panel models. *Sociol Methodol.* (1977) 8:84–136. 10.2307/270754

[14] Bentler P. Comparative fit indexes in structural models. *Psychol Bull.* (1990) 107:238. 10.1037/0033-2909.107.2.238 2320703

[15] Tucker L Lewis C. A reliability coefficient for maximum likelihood factor analysis. *Psychometrika.* (1973) 38:1–10. 10.1007/BF02291170

[16] Hu L Bentler P. Cutoff criteria for fit indexes in covariance structure analysis: Conventional criteria versus new alternatives. *Struct Eq Modeling.* (1999) 6:1–55. 10.1080/10705519909540118

[17] Browne M Cudeck R. Alternative ways of assessing model fit. *Sociol Methods Res.* (1992) 21:230–58. 10.1177/0049124192021002005

[18] Abamecha F Tena A Kiros G. Psychographic predictors of intention to use cervical cancer screening services among women attending maternal and child health services in Southern Ethiopia: the theory of planned behavior (TPB) perspective. *BMC Public Health.* (2019) 19:434. 10.1186/s12889-019-6745-x 31023306 PMC6482500

[19] Taştekin Ouyaba A İnfal Kesim S. Women’s behaviours towards cervical cancer screening in the COVID-19 pandemic: a moderated-mediation-model based on information-motivation-behavioural skills. *J Adv Nurs.* (2023) 79:125–34. 10.1111/jan.15447 36177523

[20] Gülle B Tozduman B Çelik MMÖ. Determinants of cancer screening participation in Türkiye: a nationwide study of demographic, socioeconomic, and lifestyle factors. *BMC Public Health.* (2025) 25:2637. 10.1186/s12889-025-23847-1 40753229 PMC12317624

[21] Aweke Y Ayanto S Ersado T. Knowledge, attitude and practice for cervical cancer prevention and control among women of childbearing age in Hossana Town, Hadiya zone, Southern Ethiopia: community-based cross-sectional study. *PLoS One.* (2017) 12:e0181415. 10.1371/journal.pone.0181415 28742851 PMC5526548

[22] Yeşilfidan D Özkan M Adana F. Comparison of cervical cancer-related knowledge, attitudes, and behaviors among women in urban and rural regions. *BMC Public Health.* (2025) 25:2561. 10.1186/s12889-025-24001-7 40722016 PMC12302889

[23] Hamdiui N Stein M Van Steenbergen J Crutzen R Bouman M Khan A et al. Evaluation of a web-based culturally sensitive educational video to facilitate informed cervical cancer screening decisions among Turkish- and Moroccan-Dutch women aged 30 to 60 years: randomized intervention study. *J Med Internet Res.* (2022) 24:130–42. 10.2196/35962 36287585 PMC9647450

[24] Veeravagu T Hamdiui N Stein M Crutzen R Timen A. Barriers, facilitators, needs, and preferences in seeking information regarding cervical cancer prevention programs among Turkish, Moroccan, and Syrian immigrant women: a scoping review. *BMC Public Health.* (2025) 25:1242. 10.1186/s12889-025-22359-2 40175959 PMC11963620

[25] Ważna A Dziechciaż M Wieczorowska-Tobis K Goniewicz M Al-Wathinani A Goniewicz K. Sociodemographic and occupational predictors of health behaviors among nurses: a cross-sectional study in Poland. *BMC Nurs.* (2025) 24:1135. 10.1186/s12912-025-03786-3 40890683 PMC12400615

[26] Bhandari D Shibanuma A Kiriya J Hirachan S Ong K Jimba M. Factors associated with breast cancer screening intention in Kathmandu Valley, Nepal. *PLoS One.* (2021) 16:e0245856. 10.1371/journal.pone.0245856 33481894 PMC7822561

[27] Wollancho W Amdissa D Bamboro S Wasihun Y Tareke K Gizaw A. Determining behavioral intention and its predictors towards cervical cancer screening among women in Gomma district, Jimma, Ethiopia: application of the theory of planned behavior. *PLoS One.* (2020) 15:e0238472. 10.1371/journal.pone.0238472 33151928 PMC7644032

[28] Munpolsri P Su C Yang H Hsu T Chou Y Lin L et al. Changes in risk habits and influencing factors in the Taiwan oral cancer screening program. *PLoS One.* (2025) 20:e0320461. 10.1371/journal.pone.0320461 40531846 PMC12176207

[29] Mabotja M Levin J Kawonga M. Beliefs and perceptions regarding cervical cancer and screening associated with Pap smear uptake in Johannesburg: a cross-sectional study. *PLoS One.* (2021) 16:e0246574. 10.1371/journal.pone.0246574 33566798 PMC7875386

[30] Demeke A Deribe B Girma M Gizaw M Getachew S Unverzagt S et al. Screening attendance of breast or cervical cancers and its associated factors among 30–49 year old women in Gedeo zone, South Ethiopia: cross-sectional study. *PLoS One.* (2025) 20:e0315891. 10.1371/journal.pone.0315891 39888894 PMC11785269

[31] Akiyama M Ishida N Takahashi H Takahashi M Otsuki A Sato Y et al. Screening practices of cancer survivors and individuals whose family or friends had a cancer diagnoses—a nationally representative cross-sectional survey in Japan (INFORM Study 2020). *J Cancer Survivorship.* (2023) 17:663–76. 10.1007/s11764-023-01367-4 37041402 PMC10089820

[32] Takeuchi E Kim Y Shaffer K Cannady R Carver C. Fear of cancer recurrence promotes cancer screening behaviors among family caregivers of cancer survivors. *Cancer.* (2020) 126:1784–92. 10.1002/cncr.32701 31913499 PMC7103484

[33] Henrikson P Webber E Goddard P Scrol M Piper P Williams M et al. Family history and the natural history of colorectal cancer: systematic review. *Genet Med.* (2015) 17:702–12. 10.1038/gim.2014.188 25590981 PMC4955831

[34] O’Donovan B Mooney T Rimmer B Fitzpatrick P Flannelly G Doherty L et al. Advancing understanding of influences on cervical screening (non)-participation among younger and older women: a qualitative study using the theoretical domains framework and the COM-B model. *Health Expect.* (2021) 24:2023–35. 10.1111/hex.13346 34476875 PMC8628586

[35] Kultalahti H Heinävaara S Sarkeala T Pankakoski M. Effect of test history at ages 50-64 on later cervical cancer risk: a population-based case-control study. *Cancer Res Commun.* (2023) 3:1823–9. 10.1158/2767-9764.CRC-23-0191 37700796 PMC10494786

[36] Mei X Zhong Q Chen G Huang Y Li J. Exploring health literacy in Wuhan, China: a cross-sectional analysis. *BMC Public Health.* (2019) 20:1417. 10.1186/s12889-020-09520-9 32943017 PMC7499859

[37] Liu L Qian X Chen Z He T. Health literacy and its effect on chronic disease prevention: evidence from China’s data. *BMC Public Health.* (2020) 20:690. 10.1186/s12889-020-08804-4 32410604 PMC7227325

[38] Baccolini V Isonne C Salerno C Giffi M Migliara G Mazzalai E et al. The association between adherence to cancer screening programs and health literacy: a systematic review and meta-analysis. *Prevent Med.* (2021) 91:106927. 10.1016/j.ypmed.2021.106927 34954244

[39] Uncu F Evcimen H Çiftci N Yıldız M. Relationship between health literacy, health fatalism and attitudes towards cancer screenings: latent profile analysis. *BMC Public Health.* (2025) 25:2056. 10.1186/s12889-025-23277-z 40462043 PMC12131588

[40] Yang H Li S Chen Q Morgan C. Barriers to cervical cancer screening among rural women in eastern China: a qualitative study. *BMJ Open.* (2019) 9:e026413. 10.1136/bmjopen-2018-026413 30872552 PMC6429857

[41] Mantula F Toefy Y Sewram V. Barriers to cervical cancer screening in Africa: a systematic review. *BMC Public Health.* (2024) 24:525. 10.1186/s12889-024-17842-1 38378542 PMC10877795

[42] Huang J Wang J Pang T Chan M Leung S Chen X et al. Does theory of planned behaviour play a role in predicting uptake of colorectal cancer screening? A cross-sectional study in Hong Kong. *BMJ Open.* (2020) 10:e037619. 10.1136/bmjopen-2020-037619 32764087 PMC7412617

[43] Dorce L Da Silva M Mauad J Domingues C Borges J. Extending the theory of planned behavior to understand consumer purchase behavior for organic vegetables in Brazil: the role of perceived health benefits, perceived sustainability benefits and perceived price. *Food Quality Preference.* (2021) 91:104191. 10.1016/j.foodqual.2021.104191

[44] Wang L Li X Kai Q Wang D. Examining the impact of perceived behavioral control and planning on closing the exercise intention-behavior gap: insights from a meta-analytic structural equation modeling study. *Psychol Sport Exerc.* (2024) 78:102822. 10.1016/j.psychsport.2025.102822 39952422

[45] Rajeh M. Modeling the theory of planned behavior to predict adults’ intentions to improve oral health behaviors. *BMC Public Health.* (2022) 22:1391. 10.1186/s12889-022-13796-4 35858885 PMC9297589

